# An Assessment of Publication Status of Pediatric Liver Transplantation Studies

**DOI:** 10.1371/journal.pone.0168251

**Published:** 2016-12-19

**Authors:** Thomas Breil, Daniel Wenning, Ulrike Teufel, Georg F. Hoffmann, Markus Ries

**Affiliations:** 1 Pediatric Gastroenterology and Hepatology, Center for Pediatric and Adolescent Medicine, Heidelberg University Hospital, Heidelberg, Germany; 2 Pediatric Neurology and Metabolic Medicine, Center for Pediatric and Adolescent Medicine, Heidelberg University Hospital, Heidelberg, Germany; Hvidovre Hospital, DENMARK

## Abstract

**Introduction:**

Pediatric liver transplantation is a highly specialized, challenging field. Selective reporting may introduce bias into evidence based clinical decision making, but the precise extent of unpublished data in pediatric liver transplantation is unknown today. We therefore assessed the public availability of completed clinical trials in pediatric liver transplantation.

**Methods:**

We determined the proportion of published and unpublished pre-registered, completed pediatric liver transplantation studies on ClinicalTrials.gov. The major trial and literature databases, i.e., clinicaltrials.gov, Pubmed, and Google Scholar were searched for publications. In addition, principal investigators or sponsors were contacted directly. STROBE criteria were applied for the descriptive analysis.

**Results:**

Out of N = 33 studies focusing on pediatric liver transplantation registered as completed until March 2014 on clinicaltrials.gov, N = 19 (58%) studies were published until February 2015, whereas N = 14 (42%) studies remained unpublished. The unpublished trials contain data from N = 2105 (35%) patients out of a total population of N = 6044 study participants. Median time-to-publication, i.e., the period from completion of the trial until public availability of the data was 23 IQR 10 to 28 months. Most pertinent key questions in pediatric liver transplantation, i.e., surgical procedures, immunosuppression, concomitant infections, and graft rejection were addressed in 48% of studies (N = 16/33), half of which were published.

**Conclusion:**

Half of the clinical trials in pediatric liver transplantation focused on key questions such as surgical procedures, immunosuppression, concomitant infections, and graft rejection. There is still a considerable amount of unpublished studies results in pediatric liver transplantation. Time from study completion to publication was almost twice as long as the 12 months mandatory FDAAA-timeline with a trend towards acceleration over time. The data should serve as a baseline for future progress in the field. More stringent publication of completed trials and focused multicenter research should be encouraged.

## Introduction

Evidence-based-decision making in clinical medicine is contingent upon the complete availability of clinical trial results. For the benefit of patients, robust clinical evidence is particularly important in high risk and relatively rare constellations, such as liver-transplantation. The first liver transplantation in a child was conducted in 1963 [1]. Initially the procedure was associated with high morbidity and mortality, long-term survival rates after pediatric transplantation were 11%-39% [2, 3]. Over the years the outcome of pediatric liver transplantation has improved significantly with five year survival rates of 90% and five year long-term graft survival rates of >80% [4, 5]. Thanks to improved surgical techniques, to a better perioperative intensive care-setting and surveillance, and especially due to steadily improved tailored immunosuppressive therapy [6]. Specifically, the use of cyclosporine and tacrolimus were milestones in the development for a better outcome [7]. While there have been substantial improvements in the field of pediatric liver transplantation, there is still room for further optimization which makes the availability of all clinical trials results including negative data particularly important.

It is generally known that not all clinical trials are published [8]. The precise degree of unpublished studies in pediatric liver transplantation is unknown. The knowledge of the unknown is helpful to critically interpret the medical literature currently available because it allows estimating the present degree of uncertainty and, most importantly, would serve as a benchmark for future comparisons with the goal to improve completeness. Timely availability of clinical study results allows either optimizing medial practice quickly or avoiding unnecessary exposure of subjects to clinical research. In order to quantitate the unknown and the time-to-publication, we assessed a) the public availability of results of completed, registered clinical studies in pediatric liver transplantation and b) the time to publication after completion of the trials.

## Methods

### ClinicalTrial.gov query

**Fig 1:**
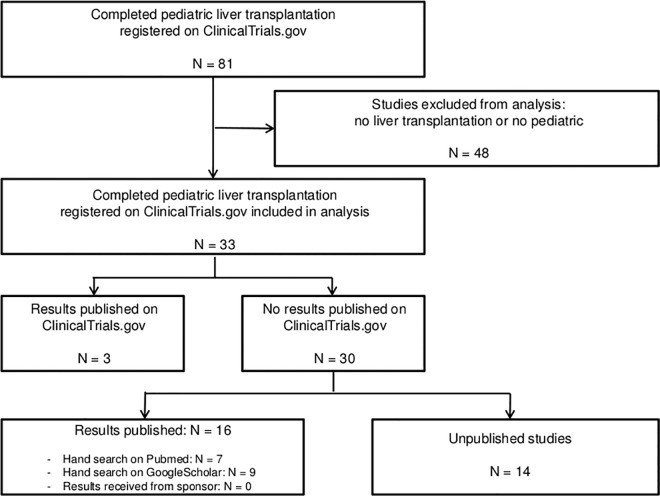
Identification of published and unpublished pediatric liver transplantation clinical trials registered on ClinicalTrials.gov: study flow diagram.

We searched ClinicalTrials.gov with the key word ‘liver transplantation’ and restricted the search to completed trials in children. Close of database was February 3, 2015. A trial was considered as published when results were presented in the ClinicalTrials.gov record or published in a peer-reviewed journal. The validity of the search results were verified manually, i.e. non-pediatric studies and studies that did not involve liver transplantation were excluded (flowchart Fig 1).

### Publication search

When the ClinicalTrial.gov record did not contain results we searched PubMed and Google Scholar for the trials in question. Publication records until February 3, 2015 were considered. Search terms for PubMed and Google Scholar included identifiers such as the NCT number, other study ID numbers listed in the ClinicalTrials.gov record, the investigated intervention, ‘liver transplantation’ or the specified condition, and details of the study design (e.g., ‘double blind’, ‘randomized’, or inclusion and exclusion criteria). If we could not find published results of registered competed trials, we contacted the principal investigator or sponsor.

### Time to publication

Time to publication for published studies was calculated as the number of months from the completion date of the trial and the publication of results either on ClinicalTrials.gov or in a peer-reviewed Journal.

### Statistical analysis

The following continuous or categorical variables were analyzed: NCT number, study title, gender, age, study phase, study type, study design, condition, intervention, recruitment status, primary completion date and completion date, availability of study results, publication date, time to publication, sponsor, and funding source. Standard methods of descriptive statistics were applied. Missing data were not imputed. All calculations were performed with SAS Enterprise Guide version 5.1 (SAS, Cary, NC, USA). STROBE criteria were respected (S1 Text).

## Results

**Fig 2:**
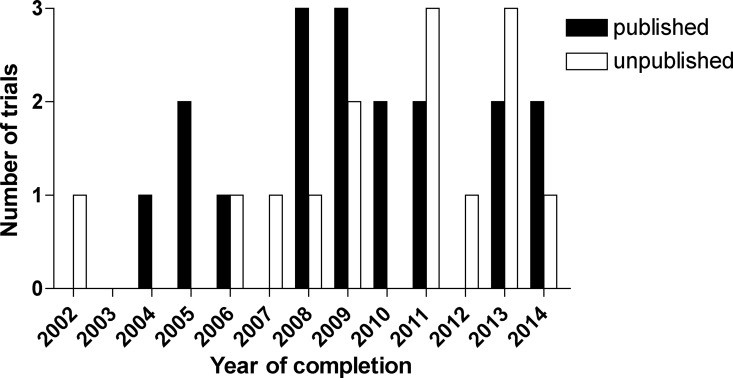
Published and unpublished pediatric liver transplantation clinical trials: number of trials by year of completion.

N = 33 studies on pediatric liver transplantation were registered as completed on clinicaltrials.gov. The year of completion ranged from 2002 to March 2014. All except one study were completed more than 12 months before database lock; close of database was February 3, 2015. N = 19 (58%) studies were published and N = 14 (42%) studies were unpublished (Figs 1 and 2, Table 1, S1 Table).

**Table 1:** Gender, age groups, trial phases, funding, type of studies

Gender		Published Studies(N = 19)	Unpublished Studies(N = 14)	Total(N = 33)
Gender				
	Male			
	Female	1		1
	Both	18	14	32
Age groups				
	Child	4	1	5
	Child/Adult	1	3	4
	Child/Adult/Senior	14	10	24
Funding				
	Industry	5	5	10
	Industry/Other	1	1	2
	NIH	4	2	6
	Other	7	6	13
	Other/industry	1		1
	Other/NIH	1		1
	U.S. Fed/NIH			
Phases				
	Phase 0			
	Phase 1	1	1	2
	Phase 1 /Phase 2			
	Phase 2	2		2
	Phase 2/Phase 3			
	Phase 3	4	1	5
	Phase 4	3	2	5
Study Type				
	Interventional	10	8	18
	Observational	9	6	15
	Randomized	7	3	10
	Non-randomized	12	11	23

**Fig 3:**
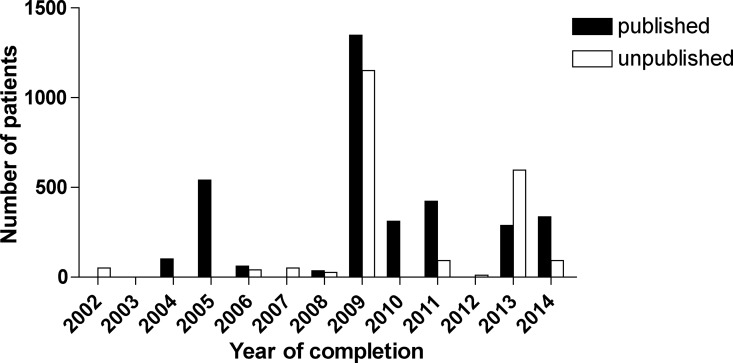
Published and unpublished pediatric liver transplantation clinical trials: number of patients by year of completion.

N = 6044 patients were enrolled in registered and completed liver transplantation clinical trials (N = 32 studies with available information). N = 3939 (65%) patients were enrolled in the published trials (N = 19 trials), whereas N = 2105 (35%) patients were enrolled in the unpublished trials (N = 13 trials with available data). The median size of the published trials was 85 IQR 19 … 300 (range 1 to 1235, N = 19) patients. On the other hand, n the unpublished trials, the median number of enrolled patients was 41 IQR 25 … 92 (range 9 to 850, N = 13) (Fig 3).

Of the published studies, one study tested a medical device, 8 studies investigated drugs, one study a gene therapy, and two studies procedures. Two trials were classified as “other“.

In the unpublished group, two trials involved biologicals, two medical studies devices, and 5 studied drugs. One study was classified as “other” (Tables 1 and 2).

**Table 2:** Conditions investigated in the published and unpublished studies in alphabetical order (N = 33). Individual studies are listed in the S1 Table.

Published studies (N = 19)	Unpublished studies (N = 14)
Candidiasis	Biliary Stricture
Heart Transplantation / Kidney Transplantation / Liver Transplantation / Lung Transplantation	Cytomegalovirus Infections
Hemophilia / Hemophilia B	Hepatitis B/Liver Transplantation
Hepatitis B, Chronic	Immunosuppression / Hepatocellular Carcinoma / Liver Transplantation / Post-operative Complications
Hepatitis B / Cirrhosis / Acute Liver Failure / Hepatocellular Carcinoma	Influenza
Hepatocellular Carcinoma / Liver Transplantation	Kidney Transplantation / Liver Transplantation / Heart Transplantation
HIV Infections / Kidney Disease / Liver Disease	Liver Diseases
Hypercholesterolemia, Familial	Liver Disease / Lymphoproliferative Disorders
Influenza	Liver Transplantation (N = 3)
Kidney Disease / Liver Disease / Pancreas Disease	Liver Transplantation / Chronic Kidney Disease
Liver Diseases	Solid Organ Transplant
Liver Transplantation (N = 2)	Virus Disease
Liver Transplantation / Infection	
Major Surgical Procedures	
Osteoporosis / Liver Transplantation / Fractures	
Patients Cannulated With Both PICC and CICC. The Rapidly Fluctuating Hemodynamics During LT.	
Pediatric Liver Transplanted Recipients / Healthy Donors	
Portopulmonary Hypertension / Pulmonary Arterial Hypertension / Pulmonary Hypertension	

Seven out of ten randomized studies and 12 out of 23 non-randomized studies were published.

### Time to publication

**Fig 4:**
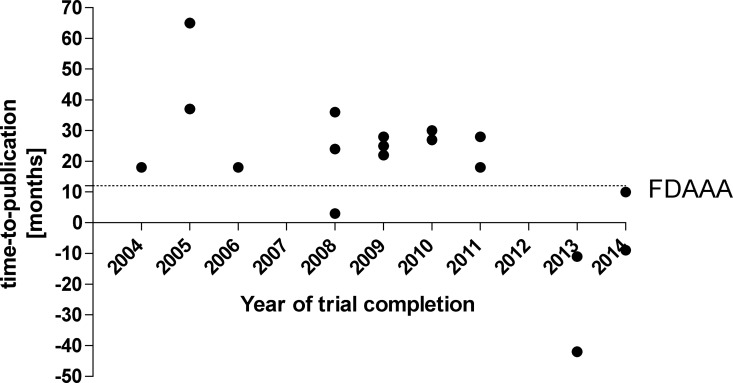
Time-to-publication of pediatric liver transplantation clinical trials. “FDAAA” indicates the timeline mandated by the Food and Drug Administration Amendments Act of 2007.

Time to publication, i.e. the period from completion of the trial until public availability of the data was 18.1 SD 22.8 (median 23 IQR 10 to 28, range -42 to 65, Fig 4) months. Three studies were published before the official completion date. Of the seven randomized published studies, four had positive results, 2 were equivocal, and 1 had negative results.

Time to publication distributions in these groups did not show a clear and consistent pattern. Two randomized studies remained unpublished. The sample sizes were too small for formal statistic comparisons.

## Discussion

We found a high degree of long-term unpublished clinical trials in pediatric liver transplantation: out of N = 33 studies registered as completed until 2014 on clinicaltrials.gov, N = 19 (58%) studies were published and N = 14 (42%) studies were unpublished. The unpublished trials contain data from N = 2105 (35%) patients out of an overall population of N = 6044 study participants. It is somewhat reassuring that most (i.e., 7/10) randomized trials were published. As a contrast, the results only nearly half of non-randomized studies are publically available. The precise extent of overall reporting of clinical trial results varies. As an example, in a recently published analysis, Anderson reported that only 38.3% of all terminated or completed clinical studies registered on clinicaltrials.gov were published [9]. On the other hand, Jones et al found that the results of 71% of registered clinical trials with more than 500 participants were available to the public [10] which is similar to the situation for completed phase III pediatric epilepsy trials, of which 24% remained unpublished [11]. This high number of unreported, completed clinical studies may distort the body of evidence on pediatric liver transplantation in the medical literature and impedes a holistic informed assessment of a given intervention in a particular circumstance. Without data transparency to the medical and scientific community, especially when negative results may not have been published, patients may be exposed to unnecessary interventions or unnecessary research risks if the same research question is being asked repeatedly. The median time to publication of trial results was relatively long with 23 months, however, there was a trend towards acceleration of reporting after the FDA Amendment Act (FDAAA) of 2007 which mandates the publication of a completed clinical trial within one year [12]. Timely availability of trial results helps to quickly adapt current practice of medicine for the benefit of patients. The pattern of sponsors for published and unpublished studies was mixed, this is in alignment with clinical practice, as many clinical studies run in tertiary referral centers. There was no bias towards industry funded studies in the published and unpublished groups (Table 1). Most published as well as unpublished studies were phase 3/4 trials. Both published and unpublished studies consisted of observational and interventional trials in a similar proportion. There are several initiatives that encourage timely publication of completed clinical trials. Besides of the FDAAA which has legal permutations within the jurisdiction of the United States, the International Committee of Medical Journals Editors encourages timely publication of results in clinical trial registries before the publication in a member journal [13]. In addition, the AllTrials initiative (www.alltrials.net), launched a petition that all past and present results of clinical trials including full methods and negative data should be published. This call is being currently supported by 622 public and private stakeholders involved in healthcare and science worldwide and was signed by 87,956 individuals by 24 April 2016 [14].

In our opinion, important questions to be addressed in pediatric liver transplantation are 1) surgical techniques and procedures, 2) the choice of appropriate immunosuppression, and, as a consequence of the latter, 3) early recognition and better management of concomitant infections and the risk of graft rejection as well as side effects of the medication. Studies registered in clinicaltrials.gov address these issues only in part, i.e. there are 16/33 (= 48%) studies investigating the above three key questions, half of them (N = 8) are published (Table 1). However, despite the significance of the subject there are overall only few active studies to-date. Therefore we would encourage more clinical research addressing the above questions, ideally—as the overall number of patients seen in a single center are usually low—in aligned multicenter projects, such as the recently inaugurated Cooperative EuRopean Paediatric TransplAnt INitiative LIver registry (http://www.certainli-registry.eu/). Other important questions in pediatric liver transplantation are donor organ allocation and prioritization. This would be difficult to address in randomized clinical trials. The opportunity likely lies in alternative approaches, e.g., quality improvement networks such as ImproveCareNow in pediatric inflammatory bowel disease [15].

### Limitations

This study has several limitations. We did not investigate other clinical trials databases, because ClinicalTrials.gov is considered the largest and most important clinical trial registry. Only registered clinical trials could be analyzed because the existence of non-registered trials is not transparent to us. In order to avoid a clinical trial being erroneously classified as unpublished, we conducted a semantic literature search in PubMed and GoogleScholar. Other databases such as EMBASE and Cochrane Library and Science Citation Index were not considered; we contacted investigators and sponsors. Some clinical studies were not strictly limited to liver transplantation but included also transplantation of other organs. We included these trials because the data are relevant to our study question, and the pattern of distribution of these subgroups was similar between the published and unpublished trials. We did not formally assess whether the content of publications was congruent with the original research questions and the pre-specified statistical analysis plan since this information was not available in the public domain for all studies. The underlying assumption of this analysis is that the data provided on ClinicalTrials.gov are accurate and complete as mandated by the FDAAA [12]. Our analysis does not take into account database analyses and clinical analyses from consortia such as studies in pediatric liver transplantation or Eurotransplant registry which may focus on important quality improvement efforts.

## Conclusion

There is still a considerable amount of selective reporting and, in part, long time to publication in pediatric liver transplantation indicating that the current body of evidence may be distorted. Not all pertinent contemporary key questions were addressed exhaustively. The data serve as baseline for the monitoring of future progress in the field.

## Supporting Information

S1 Table Individual listing of published and unpublished completed clinical studies investigating pediatric liver transplantation. Close of database: February 3, 2015
(DOCX)

S1 Text STROBE Statement—Checklist of items that should be included in reports of cross-sectional studies (DOC)
