# Supplementary material for: An Assessment of Publication Status of Pediatric Liver Transplantation Studies
Source: PLoS One. 2016 Dec 19;11(12):e0168251. doi: 10.1371/journal.pone.0168251 (PMC5167264; doi:10.1371/journal.pone.0168251)
Supplement: S1 Table — Close of database: February 3, 2015 (DOCX) [file pone.0168251.s001.docx]

**Supplementary table:** individual listing of published and unpublished completed clinical studies investigating pediatric liver transplantation.

Close of database: February 3, 2015

| **Published studies (n=19)** | |
| --- | --- |
| **Title** | **NCT number** |
| Study Evaluating Liver Transplantation in Haemophilia Patients in Spain | NCT00927992 |
| Efficacy and Safety of Basiliximab, Cyclosporine/Cyclosporine Microemulsion, and Steroids in Pediatric de Novo Liver Transplant Recipients Avoiding | NCT00149890 |
| A Study of a Modified-Release Tacrolimus Based Immunosuppression Regimen in Stable Pediatric Liver Transplant Patients | NCT00282256 |
| Antiviral Activity of Entecavir in Patients Receiving Liver Transplant Due to Chronic Hepatitis B Virus Infection | NCT00395018 |
| Liver Transplantation With ADV-TK Gene Therapy Improves Survival in Patients With Advanced Hepatocellular Carcinoma | NCT00300521 |
| Comparison of Central Venous Pressures (CVPs) Measured From Peripherally Inserted Central Catheters (PICC) and CICC in Liver Transplantation Recipients | NCT00988871 |
| The Effect of Zoledronic Acid on Bone Density in Liver Transplant Patients | NCT00114556 |
| Prevention of Recurrent Hepatitis B After Liver Transplantation | NCT00059267 |
| Living Donor Liver Transplantation in Children | NCT02124382 |
| A Study to Assess the Safety and Efficacy of Treprostinil to Facilitate Liver Transplantation in Patients With Portopulmonary Hypertension | NCT01028651 |
| Comparison of Two Treatments to Prevent Invasive Fungal Infections in Patients Who Have Received Liver Transplants | NCT00001107 |
| TachoSil Paediatric Liver Trial (TC-019-IN) | NCT00365248 |
| Influenza Vaccine in Pediatric Transplant Subjects | NCT00133510 |
| Kidney and Liver Transplantation in People With HIV | NCT00074386 |
| Endoscopic Evaluation in Transplantation Candidates | NCT00990470 |
| Effects of Donor and Recipient Genetic Expression on Heart, Lung, Liver, or Kidney Transplant Survival | NCT00531921 |
| Remote Ischemic Preconditioning In Abdominal Organ Transplantation | NCT00975702 |
| Bone Marrow Stem Cells as a Source of Allogenic Hepatocyte Transplantation in Homozygous Familial Hypercholesterolemia | NCT00515307 |
| Severe Complications Triplicate the Costs of Major Surgical Procedures | NCT00855387 |
| **Unpublished studies (n=14)** | |
| **Title** | **NCT number** |
| Evaluation of HepaGam Bￂﾮ in Combination With Antiviral Treatment in Hepatitis B Liver Transplant Patients | NCT00722332 |
| MMF After Pediatric Liver Transplantation | NCT00367146 |
| An Observational Study of Valcyte (Valganciclovir) in D+/R- Liver Transplant Recipients | NCT02062294 |
| Comparison of Immunosuppression Protocols After LTx in Children | NCT00195988 |
| Drug Use Investigation for IMURAN (Azathioprine) Tablet (Hepatic Transplantation) | NCT01390766 |
| Liver Transplantation Results in Hepatocellular Carcinoma Patients With Immunosuppression Without Steroids | NCT01137084 |
| Effect of Rapid Transfusion With Fluid Management System 2000ￂﾮ (FMS) | NCT01448343 |
| Gene Expression in Liver Allograft Rejection and Recurrent Hepatitis C | NCT01428700 |
| This Study is to Ascertain if Prolonged Release Tacrolimus (FK506E - MR4) is Safe and Effective When Used in the Long Term and in Combination With Other Immunosuppressive Drugs in Patients Who Have Received a Transplant | NCT02118896 |
| Detection and Cytotoxic T Lymphocyte Therapy of Post-Transplant Lymphoproliferative Disorder After Liver Transplant | NCT00063648 |
| Collection of Liver Tissue for Virologic Studies | NCT00005936 |
| Fully Covered Self-expandable Metal Stents (FCMS) in Benign Biliary Strictures | NCT02105181 |
| Assess the Risk of Solid Organ Transplant Rejection Following Vaccination With Pandemrix￢ﾄﾢ in UK | NCT01715792 |
| Transition From Hospital to Home in Solid Organ Transplant (SOT) | NCT00907023 |
